# What the changes in sleep architecture tell you about cognitive decline—an editorial

**DOI:** 10.1093/sleep/zsae180

**Published:** 2024-08-08

**Authors:** Kwang-Youn A Kim

**Affiliations:** Department of Preventive Medicine, Northwestern University Feinberg School of Medicine, Chicago, IL, USA

Research has shown that sleep disorders such as obstructive sleep apnea are linked to increased all-cause mortality and cardiovascular diseases [1]. Milder forms of sleep disruption such as compromised sleep quality are similarly associated with health issues such as metabolic disorders [2, 3] and cognition in older adults [4]. Given the preponderance of adults in the modern world complaining of poor sleep [5], it is fitting that Wang et al. [6] investigated the effect of the changes in sleep architecture of healthy older men as they age.

The architecture of sleep can be broadly broken down into rapid eye movement (REM) sleep, and non-REM sleep. The non-REM sleep is further composed of stages N1, N2, and N3, where each of these initial sleep stages lasts anywhere between 1 and 40 minutes with lengthening of certain stages (e.g. N2) as the night progresses [7]. Sleep metrics, such as sleep onset latency, sleep fragmentation, and the time spent in each of the stages, also evolve in complex ways as one gets older [8]. Cognitive functions have been shown (in some cases inconclusive) to be associated with these changes, where longer N2 and REM sleep were positively associated with better cognition in healthy adults [5].

Wang et al. [6] used sleep data collected from a prospective study titled Osteoporotic Fractures in Men (MrOS), investigating risk factors associated with osteoporosis in men across six clinical centers in the United States [9]. In its ancillary study, 3135 men were recruited to the MrOS Sleep study [10, 11]. Among the participants who made two visits—first in 2003–2005 and second in 2009–2012—978 patients completed polysomnography (PSG) recording and completed at least one of the cognitive function tests at visit 2. The median follow-up time between two visits was 6.5 years (*SD* = 0.7 years).

The MrOS ancillary study collected sleep data via an unattended PSG for one night. Trained individuals visited each participant’s home and collected the following key measurements: period leg movement index, apnea-hypopnea index, total sleep time, duration of wakefulness after sleep onset, and percentage of sleep time spent in N1, N2, N3, and REM sleep. The authors calculated the rate of change from visit 1 to visit 2 by taking the difference and dividing the number of years that elapsed between the two visits.

Wang et al. [6] found that the total sleep duration markedly declined with age. When total sleep was further broken down into stages, however, some stages decreased while others increased with age: duration and percentage of N1 sleep increased, duration (but not percentage) of N2 and REM sleep decreased, and duration and percentage of N3 sleep decreased significantly. The authors then analyzed if the changes in the stages of sleep were associated with cognitive decline, measured by the Modified Mini-Mental State Examination (3MS) and the Trail Making Test—Part B (Trails B), by running logistic regression models. To account for any effect of confounding variables, the models adjusted for demographics and variables such as alcohol intake, physical activity level, and medication use, among others. They showed that men with a relatively lower increase in N1 stage (25th to 75th percentile) experienced less cognitive decline compared to men with the highest increase in N1. On the other hand, when participants showed a sharp decrease in the N2 stage at visit 2, it was associated with cognitive decline on the Trails B, which is consistent with the beneficial role of N2 sleep in cognitive function [12]. Changes in N3 and REM sleep did not show any significant association with cognitive function.

It is peculiar that men who showed the mildest decrease in N1 or even minimal increase, over the course of 6.5 years, did not show further benefit compared to men who were in the 25–75th percentile. Wang et al. [6] posit that this group of men exhibited higher N1 sleep at baseline, which is negatively associated with cognitive ability, so it is possible that the benefit of minimal increase in N1 is overshadowed by the negative effect of longer N1 at baseline.

It is important to note that while Wang et al. [6] have shown the association between sleep and cognitive decline with great granularity, the study was conducted with a population of older and healthier men. This makes it more difficult to generalize its findings to women, younger adults, and an ethnically more diverse population. There may also be factors that were not considered in the statistical models. For example, environmental factors such as stress were not included in the analysis, which were shown to be related to the sleep cycles in other studies [13]. Wang et al. [6] also failed to show an association between N3 and cognitive function, which was shown to be associated with incident dementia in a recent study [14].

This study paves the way for important sleep research in the future. The longitudinal nature of measuring sleep stages and cognitive function reveals a more precise relationship between the interplay of sleep and cognitive function. Building on this knowledge, future studies could benefit from the dynamic nature of sleep by analyzing its progression of the four stages throughout the night. Coupling this with the strengths of longitudinal data collection, we will be closer to understanding the true architectural changes in sleep and how these changes are associated with cognitive decline. This would be particularly more relevant with an older population, since sleep disruption is more frequent and thus makes it more difficult to progress through all sleep cycles.
